# mAb CZP-315.D9: An Antirecombinant Cruzipain Monoclonal Antibody That Specifically Labels the Reservosomes of *Trypanosoma cruzi* Epimastigotes

**DOI:** 10.1155/2014/714749

**Published:** 2014-01-23

**Authors:** Cassiano Martin Batista, Lia Carolina Soares Medeiros, Iriane Eger, Maurilio José Soares

**Affiliations:** Laboratório de Biologia Celular, Instituto Carlos Chagas/Fiocruz, Rua Professor Algacyr Munhóz Mader 3775, Cidade Industrial, 81350-010 Curitiba, PR, Brazil

## Abstract

Reservosomes are large round vesicles located at the posterior end of epimastigote forms of the protozoan *Trypanosoma cruzi*, the etiological agent of Chagas disease. They are the specific end organelles of the endocytosis pathway of *T. cruzi*, and they play key roles in nutrient uptake and cell differentiation. These lysosome-like organelles accumulate ingested macromolecules and contain large amounts of a major cysteine proteinase (cruzipain or GP57/51 protein). Aim of this study was to produce a monoclonal antibody (mAb) against a recombinant *T. cruzi* cruzipain (TcCruzipain) that specifically labels the reservosomes. BALB/c mice were immunized with purified recombinant TcCruzipain to obtain the mAb. After fusion of isolated splenocytes with myeloma cells and screening, a mAb was obtained by limiting dilution and characterized by capture ELISA. We report here the production of a kappa-positive monoclonal IgG antibody (mAb CZP-315.D9) that recognizes recombinant TcCruzipain. This mAb binds preferentially to a protein with a molecular weight of about 50 kDa on western blots and specifically labels reservosomes by immunofluorescence and transmission electron microscopy. The monoclonal CZP-315.D9 constitutes a potentially powerful marker for use in studies on the function of reservosomes of *T. cruzi*.

## 1. Introduction

The kinetoplastid protozoan *Trypanosoma cruzi* is the etiological agent of Chagas disease, which affects about eight million people in the 18 countries in which it is endemic, mostly in Latin America [1, 2]. This parasite has a complex life cycle, with two developmental stages in the insect host (replicative epimastigotes and infective metacyclic trypomastigotes) and two stages in mammalian hosts (replicative intracellular amastigotes and infective bloodstream trypomastigotes). Macromolecule endocytosis plays an important role in this flagellate protozoan, allowing survival in the very different environments it colonizes. The endocytosis pathway has been elucidated mainly in epimastigote forms: molecules enter the cells via the flagellar pocket and cytostome, both located in the anterior region of the cell and accumulate in the reservosomes, the end compartments of the endocytosis pathway [3–6].

Reservosomes are large round vesicles located at the posterior end of *T. cruzi* epimastigotes [7]. The lack of molecular markers for cytoplasmic compartments in this parasite makes it difficult to clarify all the functions of reservosomes, which have characteristics typical of prelysosomes, lysosomes, and recycling compartments [8]. Subcellular localization [9] and proteomics [10] experiments have shown reservosomes to contain large amounts of a cysteine proteinase, known as cruzipain [11] or GP57/51 [12]. The native GP57/51 has been isolated from epimastigotes and used to generate a monoclonal antibody (mAb) [13]. Subcellular localization experiments demonstrated the presence of this protein in vesicles of the endosomal/lysosomal system and close to the flagellar pocket [12, 14]. At about the same time, the native cysteine proteinase (cruzipain) was isolated and characterized [11, 15]. A monospecific rabbit polyclonal antibody against this protein labeled reservosomes, the membrane lining the cell body and flagellum, the inside of the flagellar pocket, and even the cytostome [16]. Thus, no antibody directed against cruzipain has yet been reported to label reservosomes specifically, despite the accumulation of the enzyme in this organelle.

We report here the characterization of a mouse monoclonal antibody (mAb CZP-315.D9) against recombinant *T. cruzi* cruzipain (TcCruzipain) that specifically recognizes reservosomes. This mAb has potential as a powerful molecular marker for studies on the function of this organelle.

## 2. Materials and Methods

### 2.1. Ethics Statement

Experiments involving animals were approved by the Ethics Committee of Fiocruz (Protocol P-47/12-3 with license number LW-15/13).

### 2.2. Reagents

Polyethylene glycol (PEG), phenylmethylsulfonyl fluoride (PMSF), l-*trans*-epoxysuccinyl-l-leucylamido-(4-guanidino)-butane (E-64), alkaline phosphatase (AP)-conjugated goat anti-mouse or goat anti-rabbit antibodies, mouse anti-histidine antibody, rabbit anti-protein A antibody, bromophenol blue, *β*-mercaptoethanol, bovine serum albumin (BSA), Dulbecco's modified Eagle's medium (DMEM), HT (hypoxanthine and thymidine) medium, HAT (hypoxanthine, aminopterin, and thymidine) medium, and Roswell Park Memorial Institute-1640 (RPMI-1640) medium were purchased from Sigma Co. (St. Louis, MO, USA). Transferrin-Alexa 633, horse-radish peroxidase (HRP-) goat anti-mouse IgG (H+L), Hoechst 33342, goat anti-mouse antibodies coupled to AlexaFluor-488 or AlexaFluor-594, goat anti-rabbit antibody coupled to AlexaFluor 594, Bench Mark prestained Protein Ladder, and Bench Mark Protein Ladder were purchased from Life Technologies-Invitrogen Co. (Carlsbad, CA, USA). Alu-Gel-S adjuvant was purchased from Serva Electrophoresis GmbH Co. (Heidelberg, Germany). Fetal calf serum (FCS) was purchased from Cultilab Ltda (Campinas, SP, Brazil). Isopropylthio-*β*-galactoside (IPTG) was purchased from Anresco Laboratories Inc. (San Francisco, CA, USA). SureBlue TMB Substrate was purchased from Kirkegaard and Perry Laboratories (KPL, Gaithersburg, MD, USA). Bradford solution was purchased from BIO-RAD (Hercules, CA, USA).

### 2.3. Parasites

Cultured epimastigotes of *T. cruzi* clone Dm28c [17] were maintained at 28°C by weekly passages in liver infusion tryptose (LIT) medium [18] supplemented with 10% heat-inactivated fetal calf serum (FCS). For TcCruzipain cloning, DNA was isolated by phenol-chloroform extraction [19], from three-day-old cultures of epimastigotes.

### 2.4. Construction and Purification of Recombinant TcCruzipain Protein

The whole gene encoding *T. cruzi* cruzipain (TcCruzipain, 1404 bp, gene ID Tc00.1047053507603.260) was used to design primers (Forward: 5′-ATGTCTGGCTGGGCTCGTGCGCTG-3′ and Reverse: 5′-TCAGAGGCGACGATGACGGCTGTGGGTA-3′) with recombination sites (attBs) for use on the Gateway cloning platform (Life Technologies-Invitrogen, USA). *Escherichia coli* strain C43+ was used for recombinant protein production (TcCruzipain + pDEST17 vector expressing a histidine tag), which was induced by incubating the cell culture for 7 h with 1 mM IPTG. The production of the recombinant protein (50 kDa TcCruzipain + 6 kDa histidine tag) was confirmed by western blotting with a probe directed against the histidine tag, and the recombinant protein was purified from the polyacrylamide gel by elution.

### 2.5. Construction of Recombinant Cruzipain Domains

The whole cruzipain gene was used for domain analysis by pFAM software (Sanger Institute, Cambridge, UK). Cruzipain has three protein domains: pre-pro (aminoacids 38–94), catalytic (aminoacids 123–335), and C-terminal extension (aminoacids 337–417). The nucleotide sequence encoding each protein domain was used to design specific primers, as follows: (a) pre-pro (nucleotides 1 to 368), Forward: 5′-ATGTCTGGCTGGGCTCGTGCG-3′ and Reverse: 5′-CGCGCCCAACTACCTCAACCTTCAC-3′; (b) catalytic (nucleotides 369 to 1005), Forward: 5′-CCCGCGGCAGTGGATTG-3′ and Reverse: 5′-CACCGCAGAGCTCGCCTCCTCC-3′; (c) C-terminal extension (nucleotides 1011 to 1404), Forward: 5′-GGTCCCGGTCCCACTCCTGAGCCA-3′ and Reverse: 5′-TCAGAGGCGGCGATGACGG-3′. Primers had recombination sites (attBs) for use on the Gateway cloning platform (Life Technologies-Invitrogen, USA). *Escherichia coli* strain C43+ was used for recombinant protein production (TcCruzipain protein domains + pDEST17 vector expressing a histidine tag), which was induced by incubating the cell culture for 4 h with 1 mM IPTG. Production of recombinant proteins was confirmed by western blot with a probe directed against the histidine tag.

### 2.6. Monoclonal Antibody Production

Three male BALB/c mice (30–45-days old) received four intraperitoneal doses of 20 *μ*g TcCruzipain + Alu-Gel-S and a last intravenous (without Alu-Gel-S) injection, separated by intervals of one week. The animals were checked before immunization for antibody cross reactivity with protein extracts of *T. cruzi* epimastigotes (preimmune serum) by western blot assay.

The spleen of a TcCruzipain-reactive mouse was used in a cell fusion protocol [20]. Spleen cells were obtained by filtration, centrifugation, and washing and were fused with Ag8XP3653 myeloma cells (generously supplied by Dr. Carlos R. Zanetti, from Laboratório de Imunologia Aplicada, Universidade Federal de Santa Catarina, Brazil) in the presence of 50% polyethylene glycol (PEG). After fusion, the cells were resuspended at a density of 2.5 × 10^6^ cells/mL in RPMI medium supplemented with 20% FCS and 100 *μ*L of this suspension was added to each well of a 96-well plate. The cells were allowed to grow for 24 h at 37°C, under an atmosphere containing 5% CO_2_, and 100 *μ*L of HAT medium was then added to the cell culture. The medium was replaced every 48 h. Hybrid cells were selected over a period of 14 days, and the medium was then replaced with HT medium for an additional four days. Hybrid cells were selected and propagated in RPMI medium containing 20% FCS. Positive hybridomas were selected by indirect ELISA, western blotting, and indirect immunofluorescence (see below).

The most stable hybridoma in cryosurvival assays [20] was cloned by limiting dilution. The SBA Clonotyping-HRP System (Southern Biotech, Birmingham, USA), based on capture ELISA, was used to identify mAb isotype, in accordance with the manufacturer's instructions. Positive hybridomas and clones were cryopreserved at the Laboratório de Biologia Celular (ICC/FIOCRUZ-PR).

### 2.7. ELISA

For indirect ELISA, recombinant TcCruzipain (0.15 *μ*g/well) was adsorbed onto 96-well immunoplates (Nunc, Roskilde, Denmark) by incubation overnight at 4°C with sensitizing buffer (0.05 M sodium carbonate and sodium bicarbonate, pH 9.6). The plates were then blocked by incubation for 1 h with 5% nonfat milk powder in PBS supplemented with 0.01% Tween 20 (PBS-T). The hybridoma supernatants were added to the immunoplates and incubated for 1 h at 37°C. The plates were washed five times with PBS-T and incubated for 1 h at 37°C with HRP-conjugated goat anti-mouse IgG (1 : 4,000). The plates were then washed five times with PBS-T and immunoreactivity was visualized with the SureBlue TMB Substrate, with optical density (OD) being read at 450 nm in an EL800 ELISA reader (BioTek, Winooski, VT, USA). Only OD values higher than 0.300 were considered positive.

### 2.8. Western Blot

For expression analysis of native cruzipain on *T. cruzi* epimastigotes, total protein extracts of the parasites were prepared by resuspending PBS-washed parasites (10^9^ cells/mL) in denaturing buffer A (40 mM Tris-HCl pH 6.8; 1% SDS; 360 mM *β*-mercaptoethanol). PMSF (1 mM) and E-64 (100 *μ*M) were used as protease inhibitors. Protein content was determined in a Bradford assay [21]. The samples were resuspended in denaturing buffer B (40 mM Tris-HCl pH 6.8; 1% SDS; 360 mM *β*-mercaptoethanol; 6% glycerol; 0.005% bromophenol blue) and boiled at 100°C for 5 min. Protein extracts (15 *μ*g protein/lane) were fractionated by SDS-PAGE in 10% polyacrylamide gels and the resulting bands were transferred onto nitrocellulose membranes (Hybond C, Amersham Biosciences, England), according to standard protocols [19, 22]. Following protein transfer, the membranes were blocked by incubation with 5% nonfat milk powder/0.05% Tween-20 in PBS. The membranes were then incubated for 1 h with blocking buffer containing preimmune serum (diluted 1 : 200), antirecombinant TcCruzipain polyclonal serum (diluted 1 : 500), antirecombinant TcCruzipain hybridoma (CZP-315) supernatant, or antirecombinant TcCruzipain monoclonal antibody (mAb CZP-315.D9, diluted 1 : 100). The membrane was washed three times in 0.05% Tween-20/PBS and then incubated for 1 h with AP-conjugated rabbit anti-mouse IgG (diluted 1 : 10,000). A polyclonal antirecombinant actin (TcActin; diluted at 1 : 200) mouse serum [23] was used for normalization. The membrane was then washed three times with 0.05% Tween-20/PBS and the reactive bands were visualized with BCIP-NBT solution, as described by the manufacturer.

To verify the specificity of mAb CZP-315.D9, whole protein extracts of *E. coli* (15 *μ*g protein/lane) and purified recombinant TcCruzipain (2 *μ*g protein/lane) were fractionated by SDS-PAGE in 10% polyacrylamide gels, transferred onto nitrocellulose membranes, and incubated with either the antirecombinant TcCruzipain polyclonal serum (diluted 1 : 1000 in blocking buffer) or the mAb CZP-315.D9 (diluted 1 : 100 in blocking buffer). The membrane was washed three times in 0.05% Tween-20/PBS and then incubated for 1 h with AP-conjugated rabbit anti-mouse IgG (diluted 1 : 10,000). The membrane was then washed three times with 0.05% Tween-20/PBS and the reactive bands were visualized with BCIP-NBT solution, as described by the manufacturer.

For analysis of cruzipain domain labeling, protein extracts of *E. coli* vector (with cruzipain domains) were fractionated by SDS-PAGE (15 *μ*g protein/lane) in 10% polyacrylamide gels, transferred onto nitrocellulose membranes, and incubated with the antirecombinant TcCruzipain polyclonal serum (diluted 1 : 1000) or with the mAb CZP-315.D9 (diluted 1 : 100). The experiment then continued was described above.

### 2.9. Fluorescence Microscopy

*T. cruzi* epimastigotes were washed twice in PBS, fixed by incubation for 30 min with 4% paraformaldehyde, permeabilized by incubation for 5 min with PBS/0.5% Triton, and incubated for one hour at 37°C with preimmune serum diluted 1 : 150 in PBS pH 7.4 containing 1.5% BSA (incubation buffer), anti-TcCruzipain polyclonal serum diluted 1 : 500 in incubation buffer, anti-TcCruzipain hybridoma (CZP-315) supernatant, or antirecombinant TcCruzipain mAb (CZP-315.D9) diluted 1 : 40. Samples were washed three times with PBS and then incubated, in the same conditions, with goat anti-mouse secondary antibody coupled to AlexaFluor 488 or 594 diluted 1 : 600 in incubation buffer. The samples were washed three times with PBS, incubated for 5 min with 1.3 nM Hoechst 33342 (DNA marker), and examined under a Leica SP5 confocal laser microscope (Leica Microsystems, Wetzlar, Germany).

We further analyzed colocalization of native cruzipain in the Golgi apparatus in transfected epimastigotes expressing the *T. cruzi* Golgi marker TcHIP/AC [24]. Three-day-old culture transfected epimastigotes were washed twice in PBS, fixed for 30 min with 4% paraformaldehyde, and incubated for one hour at 37°C with anti-TcCruzipain mouse polyclonal serum (1 : 500) and rabbit anti-protein A antibody (1 : 40,000). The samples were washed three times in PBS and incubated, in the same conditions, with the secondary antibodies: goat anti-mouse antibody coupled to AlexaFluor 488 and goat anti-rabbit antibody coupled to AlexaFluor 594 (both diluted 1 : 600). Fluorescence microscopy was then carried out as described above.

### 2.10. Endocytosis Assay

*T. cruzi* epimastigotes were washed twice in PBS and then subjected to nutritional stress in PBS for 15 min at 25°C. They were then incubated for 30 min at 28°C with transferrin coupled to Alexa 633 (1 mg/mL) diluted 1 : 40. This period was long enough for the ingested transferrin to accumulate in the reservosomes [3]. For the colocalization of transferrin with native cruzipain, the fed parasites were then fixed by incubation for 30 min with 4% paraformaldehyde, permeabilized by incubation for 5 min with 0.5% Triton in PBS, and incubated with the CZP-315.D9 mAb diluted 1 : 40 in incubation buffer. The samples were washed three times in PBS and then incubated, in the same conditions, with a goat anti-mouse secondary antibody coupled to AlexaFluor 488 (1 : 600) in incubation buffer. The samples were washed three times with PBS, incubated for 5 min with 1.3 nM Hoechst 33342, and examined under a Leica SP5 confocal laser microscope.

### 2.11. Transmission Electron Microscopy

Culture epimastigotes were collected by centrifugation, washed three times in phosphate buffer (pH 7.2), and fixed for 1 h at room temperature with 0.1% glutaraldehyde + 4% paraformaldehyde in 0.1 M phosphate buffer. The cells were then washed in phosphate buffer, dehydrated in graded ethanol series, and infiltrated overnight at low temperature (−20°C) with a 1 : 1 dilution of ethanol 100%: Lowicryl K4M or Lowicryl K4M MonoStep resin (EMS, Hatfield, PA, USA). After embedding for 6 h in pure resin, the samples were polymerized for 48 h at −20°C under UV light. Ultrathin sections (70 nm) were collected on nickel grids, incubated for 30 min with 50 mM ammonium chloride in PBS (pH 7.2), and then incubated for 1 h with mAb CZP-315.D9 diluted 1 : 20 in incubation buffer. After washing in this buffer, the grids were incubated for 1 h with a rabbit anti-mouse antibody coupled to 10 nm gold particles diluted at 1 : 20 in incubation buffer. After washing in buffer and distilled water, the grids were stained for 45 min with 5% uranyl acetate and for 5 min with lead citrate and observed in a JEOL 1200EXII transmission electron microscope operated at 80 kV.

## 3. Results

### 3.1. Production, Characterization, and Specificity of the Anti-TcCruzipain Monoclonal Antibody

The *T. cruzi* cruzipain gene was amplified, cloned (as confirmed by sequencing), and expressed in *E. coli*, producing a 56 kDa recombinant protein (50 kDa of TcCruzipain sequence + 6 kDa of his-tag) that was purified and used to immunize BALB/c mice. The mouse with the most responsive and specific anti-TcCruzipain serum (as determined by western blotting and subcellular localization by indirect immunofluorescence) was chosen for fusion of splenocytes with myeloma cells. Seven positive hybridomas were detected by indirect ELISA. The most stable hybridoma (CZP-315) was used to obtain clones by limiting dilution. An IgG1 isotype (OD value: 1.105) and kappa-positive (OD value: 0.459) monoclonal antibody (mAb CZP-315.D9) was obtained after selection by indirect ELISA, western blotting, and indirect immunofluorescence assays (Table 1).

**Figure 1:**
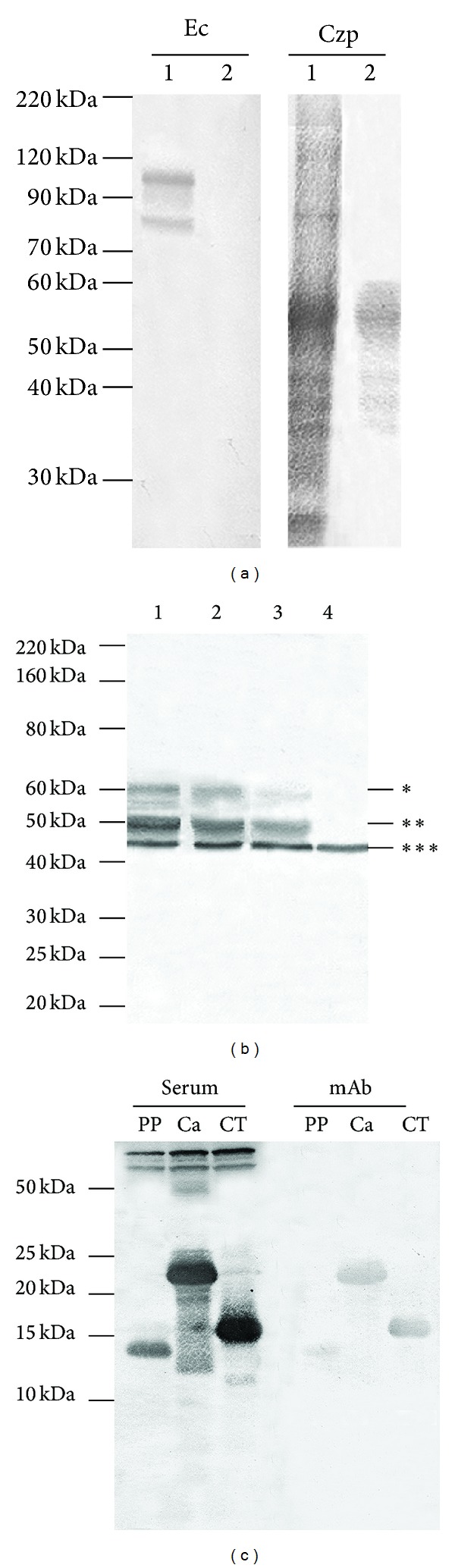
Western blot analysis of antibodies against TcCruzipain. (a) Protein extracts of *E. coli* (Ec) and purified recombinant TcCruzipain (Czp) incubated with anti-TcCruzipain polyclonal serum (lane 1) or CZP-315.D9 monoclonal antibody (lane 2). (b) Total protein extracts of *T. cruzi* epimastigotes incubated with anti-TcCruzipain serum (lane 1), CZP-315 hybridoma supernatant (lane 2), CZP-315.D9 monoclonal antibody (lane 3) or preimmune serum (lane 4). Actin was used for normalization. (c) Protein extracts of vector-bacteria containing recombinant cruzipain domains incubated with polyclonal antibodies (serum) or with mAb CZP-315.D9 (mAb) against TcCruzipain. PP: prepro domain; Ca: catalytic domain; CT: C-terminus domain. The Bench Mark Protein Ladder was used to determine molecular weights. *Immature cruzipain; **mature cruzipain; ***TcActin.

A western blotting assay was performed to compare the reactivity of the anti-TcCruzipain polyclonal serum and the mAb CZP-315.D9 to *E. coli* protein extracts and to purified recombinant TcCruzipain. The anti-TcCruzipain serum recognized three protein bands between 80 and 110 kDa in *E. coli* (Figure 1(a), Ec lane 1) and several protein bands with the recombinant TcCruzipain (Figure 1(a), Czp lane 1), but with higher reactivity to a protein band between 50 and 60 kDa, compatible with TcCruzipain (50 kDa TcCruzipain + 6 kDa histidine tag). On the other hand, the mAb CZP-315.D9 recognized no protein bands in *E. coli* (Figure 1(a), Ec lane 2) but recognized the protein band between 50 and 60 kDa in the purified TcCruzipain fraction (Figure 1(a), Czp lane 2). Furthermore, both polyclonal and monoclonal antibodies recognized three protein bands below 50 kDa in the TcCruzipain fraction.

We further assessed the specificity of the CZP-315.D9 mAb against whole-epimastigote extracts by western blotting. Both anti-TcCruzipain polyclonal serum and CZP-315 hybridoma supernatant recognized two protein bands between 50 and 60 kDa, whereas the CZP-315.D9 mAb recognized mainly the protein band with about 50 kDa. The preimmune serum did not recognize any proteins. Actin (42 kDa), used for normalization, was detected with a polyclonal anti-TcActin mouse serum (Figure 1(b)).

Western blot assay was performed to determine which cruzipain domain (pre-pro domain, catalytic domain or C-terminal extension) was recognized by the polyclonal serum and by mAb CZP-315.D9. While the polyclonal serum recognized all protein domains and crossreacted with *E. coli* (protein bands below 50 kDa, Figure 1(c)), the mAb CZP-315.D9 did not, or weakly, recognize the pre-pro domain (Figure 1(c)).

### 3.2. Localization of Cruzipain in Reservosomes and Colocalization with Ingested Transferrin

**Figure 2:**
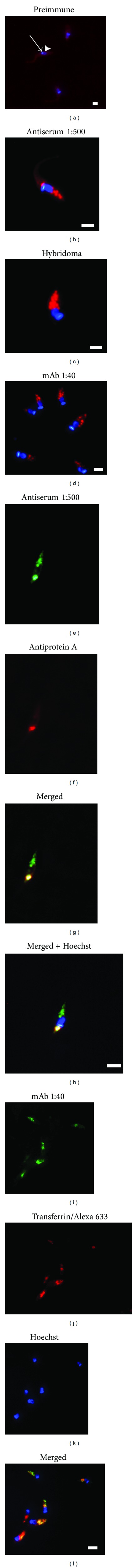
Immunolocalization of cruzipain and its colocalization with TcHIP/AC and ingested transferrin in *Trypanosoma cruzi* epimastigotes. The nucleus (arrowhead) and kinetoplast (arrow) are stained blue with Hoechst 33342. (a) Incubation with preimmune serum. (b) Incubation with anti-TcCruzipain serum. Note labeling of reservosomes and a single spot at the anterior end of the cell. (c) Incubation with CZP-315 hybridoma supernatant. Note that only reservosomes are labeled. (d) Incubation with CZP-315.D9 monoclonal antibody. Labeling is specific for reservosomes. ((e)–(h)) Colocalization of anti-TcCruzipain serum (green staining) with the Golgi marker TcHIP (red staining) in TcHIP/AC-transfected epimastigotes. ((i)-(l)) Endocytosis assay with transferrin coupled to Alexa 633 (red staining), and its colocalization with cruzipain (green staining) in epimastigotes, resulting in yellow staining. Bars = 5 *μ*m.

*T. cruzi* epimastigotes were incubated with preimmune serum, anti-TcCruzipain polyclonal serum, CZP-315 hybridoma supernatant, or mAb CZP-315.D9. As expected, no labeling was observed with the preimmune serum (Figure 2(a)). The anti-TcCruzipain polyclonal serum labeled several round spots at the posterior end of the parasites (reservosomes) and a single spot at the anterior end of the cells, lateral to the kinetoplast (Figure 2(b)), corresponding to the Golgi complex (see below). The CZP-315 hybridoma supernatant and the mAb CZP-315.D9 recognized only the round spots (reservosomes) at the posterior end of the parasites (Figures 2(c) and 2(d)).

Incubation of TcHIP/AC transfectant epimastigotes with both anti-protein-A tag and anti-TcCruzipain polyclonal sera showed colocalization of the protein A tag and TcCruzipain at a single spot at the anterior end of the cells, lateral to the kinetoplast, corresponding to the single Golgi complex of the parasites (Figures 2(g) and 2(h)). No colocalization was observed at the posterior end of the parasites, which displayed only cruzipain labeling in several round structures (reservosomes).

An endocytosis assay was performed to validate the mAb CZP-315.D9. Alexa 633-conjugated transferrin was internalized and directed to the reservosomes, where it colocalized with cruzipain labeling (Figures 2(i)–2(l)).

**Figure 3:**
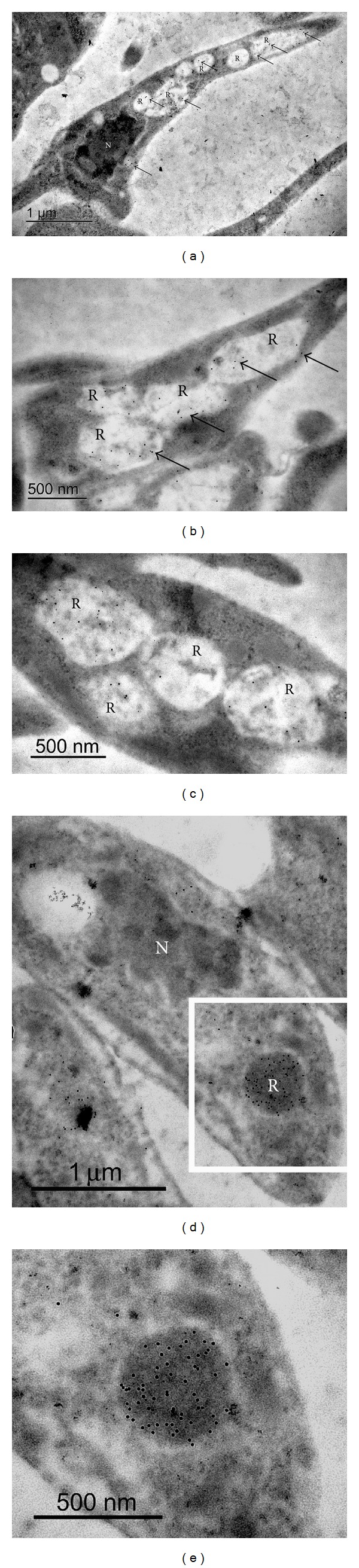
Immunolocalization of cruzipain in *Trypanosoma cruzi* epimastigotes by transmission electron microscopy. Ultrathin sections were incubated with the mAb CZP-315.D9, followed by a secondary antibody coupled to 10 nm gold particles. Note the specific gold labeling (arrows) in the reservosomes. Weaker labeling was found in cells embedded with Lowicryl K4M resin ((a)–(c)), while more intense labeling was found in the electrondense reservosomes of cells embedded with Lowicryl K4M MonoStep resin ((d)-(e)). (e) shows a high magnification of the area delimited in (d). N: nucleus; R: reservosome.

We further assessed the immunolocalization of cruzipain by transmission electron microscopy (TEM). After incubating mAb CZP-315.D9 with ultrathin sections of epimastigote forms, gold labeling was found specifically in reservosomes (Figure 3). Weaker labeling was found in reservosomes from cells embedded with Lowicryl resin, which appeared electronlucent (Figures 3(a)–3(c)). More intense labeling was found in reservosomes from cells embedded with Lowicryl MonoStep resin (Figures 3(d) and 3(e)), which appeared more electrondense.

## 4. Discussion

Reservosomes are large round vesicles at the posterior end of *T. cruzi* epimastigote forms, in which the macromolecules taken up by the parasites accumulate [3]. Reservosomes are thus specific end organelles of the endocytosis pathway of this protozoan and can be used as exclusive markers/targets for these parasites. Proteomics analyses have shown that reservosomes contain several lysosomal enzymes [10], including a major cysteine proteinase known as cruzipain [11] or GP57/51 [12]. However, the antibodies against cruzipain currently available do not specifically target the reservosomes [11, 12]. We, therefore, aimed to produce a monoclonal antibody (mAb) against recombinant cruzipain (TcCruzipain) that specifically labeled reservosomes.

Indirect immunofluorescence assays to detect cruzipain in *T. cruzi* epimastigotes showed that (a) following incubation with a polyclonal serum against TcCruzipain, labeling was restricted to the reservosomes and in a single spot lateral to the kinetoplast and (b) following incubation with hybridoma supernatant and the mAb against TcCruzipain, labeling was restricted to the reservosomes. Immunolocalization of cruzipain by transmission electron microscopy showed gold labeling specifically in reservosomes. More intense labeling in electrondense reservosomes could be due to sample preservation in different resins (Lowicryl K4M and Lowicryl K4M MonoStep). Previous antibodies against cruzipain have labeled reservosomes, the membrane lining the cell body and flagellum, the inside of the flagellar pocket, and even the cytostome [9, 12, 14, 16]. Our monoclonal antibody, therefore, appears to be a suitable tool for the specific labeling of reservosomes.

TcHIP is a marker of the Golgi apparatus of *T. cruzi* [24]. Incubation of TcHIP/AC-transfected epimastigotes with both anti-protein-A tag and anti-TcCruzipain polyclonal sera revealed colocalization of protein A and TcCruzipain in a single spot at the anterior end of the cells, close to the kinetoplast, in a region corresponding to the Golgi complex. Cruzipain is a glycoprotein that is edited in the Golgi complex and then directed to the endosomal/lysosomal system via the *trans*-Golgi network [25, 26]. Our polyclonal serum, therefore, also recognized immature cruzipain in transit through the Golgi complex, whereas the CZP-315 hybridoma and CZP-315.D9 mAb recognized the mature cruzipain in the reservosomes.

In western blot assays with whole extracts of *T. cruzi* epimastigote forms, both the anti-TcCruzipain serum and the CZP-315 hybridoma supernatant recognized two protein bands between 50 and 60 kDa, whereas the CZP-315.D9 mAb reacted strongly with a protein band at about 50 kDa. Cruzipain is produced as a 57 kDa protein, from which 6 kDa is cleaved to generate the mature cysteine protease, which thus has a molecular weight of 51 kDa (GP57/51) [12]. These data thus indicate that our CZP-315.D9 mAb recognizes mainly the mature enzyme in the reservosomes. Western blot assay to compare recognition of the anti-TcCruzipain polyclonal serum and the mAb CZP-315.D9 to purified recombinant TcCruzipain showed that the monoclonal recognized mainly a protein band between 50 and 60 kDa (50 kDa TcCruzipain + 6 kDa histidine tag), thus confirming the higher specificity of this mAb, as compared to a polyclonal antiserum. Both polyclonal and monoclonal antibodies recognized three protein bands below 50 kDa in a TcCruzipain fraction, probably due to proteolysis.

Cruzipain has three protein domains: pre-pro, catalytic, and C-terminal extension [27]. Our polyclonal serum recognized all protein domains by western blot analysis. On the other hand, mAb CZP-315.D9 recognized the catalytic domain and the C-terminal extension but did not, or weakly, recognize the pre-pro domain. This double binding can be dependent on conformational epitopes. No labeling with the pre-pro domain indicates why the mAb CZP-315.D9 does not recognize the immature cruzipain present in the Golgi complex.

An endocytosis assay was carried out with epimastigotes to validate the mAb CZP-315.D9. Transferrin ingested by the parasites was clearly colocalized with cruzipain labeling in the reservosomes. Thus, we demonstrate here, for the first time, the production of a specific mAb against reservosomal cruzipain. Monoclonal antibodies present several advantages over polyclonal sera, such as specificity, reproducibility, and ethical advantages [28]. The mAb produced in this study thus appears to be a potentially powerful molecular marker for studies on the function of this species-specific organelle, which plays an important role in the endocytosis of nutrients and cell differentiation (metacyclogenesis) in *T. cruzi* [29, 30].

## 5. Conclusions

We report here the production of a kappa-positive monoclonal IgG antibody (mAb CZP-315.D9) that recognizes recombinant *T. cruzi* cruzipain (TcCruzipain). This mAb binds mainly to a protein with a molecular weight of about 50 kDa on western blots and specifically labels reservosomes in *T. cruzi* epimastigotes by immunofluorescence and transmission electron microscopy. It thus constitutes a potentially powerful marker for use in studies on the function of these organelles.

## Figures and Tables

**Table 1:** Characterization of mAb CZP-315.D9 with the SBA Clonotyping-HRP System. Numbers are the optical density (OD) values read at 450 nm. Only OD values above 0.300 were considered positive. RPMI medium was used as a negative control.

	Ig (H+L)	IgM	IgA	IgG1	IgG2a	IgG2b	IgG3	Kappa	Lambda
Anti-TcCruzipain serum	1.207	0.852	0.585	1.24	0.816	0.996	0.19	1.125	0.33
Hybridoma 315	0.99	0.085	0.077	1.259	0.512	0.078	0.069	0.362	0.058
mAb CZP-315.D9	0.788	0.055	0.056	1.105	0.085	0.164	0.065	0.459	0.133
Medium (RPMI)	0.059	0.056	0.049	0.064	0.068	0.061	0.072	0.108	0.059
